# Implementation and Validation of Telepathology Triage at Cancer Referral Center in Rural Rwanda

**DOI:** 10.1200/JGO.2015.002162

**Published:** 2016-01-20

**Authors:** Tharcisse Mpunga, Bethany L. Hedt-Gauthier, Neo Tapela, Irenee Nshimiyimana, Gaspard Muvugabigwi, Natalie Pritchett, Lauren Greenberg, Origene Benewe, David S. Shulman, James R. Pepoon, Lawrence N. Shulman, Danny A. Milner

**Affiliations:** **Tharcisse Mpunga**, **Irenee Nshimiyimana**, **Gaspard Muvugabigwi**, Ministry of Health; **Tharcisse Mpunga**, **Bethany L. Hedt-Gauthier**, University of Rwanda; Bethany L. Hedt-Gauthier, Neo Tapela, Natalie Pritchett, **Lauren Greenberg**, **Origene Genewe**, **James R. Pepoon**, Partners In Health, Kigali, Rwanda; **Neo Tapela**, **James R. Pepoon**, **Danny A. Milner Jr**, Brigham and Women’s Hospital; **Bethany L. Hedt-Gauthier**, **David S. Shulman**, Boston Children’s Hospital; **Lawrence N. Shulman**, Dana-Farber Cancer Institute, Boston, MA; and **Lawrence N. Shulman**, Abramson Cancer Center, University of Pennsylvania, Philadelphia, PA.

## Abstract

**Purpose:**

Connecting a cancer patient to the appropriate treatment requires the correct diagnosis provided in a timely manner. In resource-limited settings, the anatomic pathology bridge to efficient, accurate, and timely cancer care is often challenging. In this study, we present the first phase of an anatomic telepathology triage system, which was implemented and validated at the Butaro District Hospital in northern rural Rwanda.

**Methods:**

Select cases over a 9-month period in three segments were evaluated by static image telepathology and were independently evaluated by standard glass slide histology. Each case via telepathology was classified as malignant, benign, infectious/inflammatory, or nondiagnostic and was given an exact histologic diagnosis.

**Results:**

For cases triaged as appropriate for telepathology, correlation with classification and exact diagnosis demonstrated greater than 95% agreement over the study. Cases in which there was disagreement were analyzed for cause, and the triage process was adjusted to avoid future problems.

**Conclusion:**

Challenges to obtaining a correct and complete diagnosis with telepathology alone included the need for immunohistochemistry, assessment of the quality of images, and the lack of images representing an entire sample. The next phase of the system will assess the effect of telepathology triage on turnaround time and the value of on-site immunohistochemistry in reducing that metric and the need for evaluation outside of telepathology.

## INTRODUCTION

Cancer is a leading cause of death and disability worldwide that accounted for 7.6 million deaths in 2008, of which 4.6 million (60%) occurred in low- and middle-income countries (LMICs).^1^ In Africa, 1.1 million new cases are projected to occur by 2020.^2^ Despite this enormous and growing burden, access to quality cancer care, including skilled personnel, diagnostic resources, and treatment resources, is limited in these settings.^3^

One gap in the complete cancer care cycle in LMICs is inadequate pathology resources as a result of extreme shortages of pathologists, deficiency of infrastructures and reagents, and poor specimen handling and storage.^4^ Before 2012 in Rwanda, histopathology services were only available at three public national referral facilities, which all faced infrastructure challenges and high workloads. Final reports often were not sufficient to affect therapy because of the lack of advanced diagnostic evaluation (eg, special stains, immunohistochemistry, molecular diagnostics), and there often were long delays in reporting.

In July 2012, the Butaro Cancer Center of Excellence (BCCOE) was established at the Butaro District Hospital in rural Rwanda in partnership with the Rwanda Ministry of Health, Partners in Health (PIH), and the Dana-Farber Brigham and Women’s Cancer Center. Oncological services for patients were developed and provided through training and support by clinicians and nursing staff from the Dana-Farber Cancer Institute. Because of limited in-country pathology capacity, unprocessed tissue specimens from Butaro were sent to Brigham and Women’s Hospital (BWH; Boston, MA) for pathology services before 2012; this required transport of tissue in fixative to Boston. Beginning in 2012, services were scaled-up on site at BCCOE, which included a fully functioning anatomic pathology laboratory and continued diagnostic support from BWH.^5^ Because of limited in-country pathology expertise, the standard at the opening of the histopathology laboratory was to send partially processed material to BWH for interpretation and to implement gradual capacity building at BCCOE, such that stained-glass pathology slides could be produced by the start of the study. After enhancement of the BCCOE pathology laboratory to include processing through staining of slides, the median time from staining to diagnosis was 32 days (range, 7 to 193 days).^6^ Although this was an improvement from the pathology services available before 2012, the time from staining to diagnosis was still longer than optimal and, in cases of emergency, often too late to affect care.

Telepathology services have been documented to bring benefits to health systems and patients by facilitating timely access to cost-effective, high-quality health care services.^3,4,7^ However, limited studies have described the use of telepathology to support cancer care in sub-Saharan Africa. In March 2013, BCCOE started piloting the use of static image telepathology, which included training of technicians, set-up of internet connections, and utilization of online sharing tools. Here, we describe our experience with the telepathology pilot from January 2014 to September 2014, including the agreement of diagnoses made via telepathology compared with traditional glass slide diagnosis and the lessons learned.

## METHODS

### Study Setting

Opened in 2011, Butaro District Hospital is a district hospital in the Rwandan health system that had a 175-bed capacity and an occupancy rate of 66% in 2013. The hospital is operated by the Ministry of Health with support from PIH, a US-based nongovernmental organization. Clinical services are provided through inpatient and outpatient departments. The hospital serves as the referral center for 19 health centers in its catchment area in the Burera district in Northern Rwanda. Before 2012, the laboratory provided services in basic hematology and chemistry, parasitology, and CD4 counts.

The BCCOE opened in July 2012, and, in the first 18 months of operations, 1,713 individuals were tested for cancer, and 1,689 were enrolled in care (data extracted from Butaro Hospital record); individuals came from all areas of the country. Histopathology services started with the inauguration of the leveraged partnership of BCCOE with BWH (described elsewhere).^6^ A pilot of telepathology services, which included a ramp-up to capacity, technical logistics, and training, started in March 2013 in parallel with standard diagnosis. Although telepathology diagnoses that are based on dynamic, real-time readings are ideal,^8^ we opted for static telepathology for this setting because of internet instability and limited bandwidth. In addition, we were seeking an intermediate solution to allow screening of cases and rapid provision of interpretation for common tumors (ie, squamous cell carcinoma), which require little ancillary testing and make up a large volume of the case load at this stage of intervention; therefore, static image telepathology was an appropriate initial choice for triaging cases and shortening the turn-around time for some cases.

### Study Design

To understand workflow and implementation challenges, we initiated the study in three segments. The first segment, considered a training segment, from January to March 2014, included mandatory upload by the technicians of all cases (12 to 16 images per case) and a review of images by only two pathologists. The goal for this first segment was to identify any issues with pathology review workflow and the quality and quantity of images. The second segment, a technical workflow segment, from April to May 2014, included no requirement for the upload of cases; each uploaded case was reviewed by two to six pathologists. The goal in the second segment was to identify any challenges for technicians in workflow and self-sufficiency in uploading cases. The third segment, the testing segment, from June to September 2014, included mandatory upload of all cases, with 12 to 16 images for each case, and each case was reviewed by a full team of six pathologists.

A histopathology laboratory technician uploaded select coded images to iPath software (http://www.ipath-network.com) and sent these images to a histopathologist at BWH for review. A preliminary feedback report was sent through the same system and took up to 24 hours, according to the availability of the histopathologist. During this study period, stained-glass slides were also sent to Boston for review, per standard of care, and the final diagnostic decision was based on the diagnosis made via traditional review of stained-glass slides.

### Triage suitability

The goal of the study was to validate the accuracy of telepathology for a subset of cases by using triage. As part of this goal, we distinguished cases as suitable for telepathology or requiring triage to Boston (ie, a telepathology diagnosis cannot be made). Specific case types and diagnoses over the duration of all phases of the study were evaluated for the potential to be confirmed by telepathology versus by requiring triage to Boston for review. Bone marrow aspirates and suspected hematologic malignancies were a priori deferred to Boston because of requirements for special stains and immunohistochemistry. In addition, pathologists who reviewed telepathology images would choose to identify a case as needing triage to Boston for any of the following possibilities: clinical history/pathologic image mismatch (eg, clinician reports a mass but the tissue images appear normal); image quality/quantity issues (eg, too few, out of focus, color off); magnification limitations (eg, need to review multiple high-power fields to find key diagnostic elements); and/or ancillary testing (eg, immunohistochemistry, special stains and so on, are needed).

### Data Collection and Analysis

The telepathology program was implemented across three project segments of training (January to March 2014), technical workflow (April to May 2014), and testing (June to September 2014). For each telepathology case uploaded to the iPath system, the final diagnosis determined by the BWH pathology department traditional read was extracted from the final report database along with sex, age, and other demographics recorded in the Butaro records. The telepathology and traditional diagnoses were classified as nondiagnostic, infectious/inflammatory, benign, or malignant.

We describe the sex, age, and cancer types for each case by testing segment. The two diagnoses (telepathology and traditional) were compared by overall percentage of agreement and κ agreement. The data for the κ statistic was weighted as follows. When there was complete agreement between the telepathology and traditional slide reading, the case was coded as 1. When there was a disagreement, the case was coded as 0, except when the one diagnosis was infectious/inflammatory and the other diagnosis was benign; then, the case was coded as 0.5 to give a small penalty for miscategorization of an infectious process as benign. Finally, cases with no final telepathology or traditional diagnosis were not included in the κ statistic, and these were reviewed to describe the reasons for nondiagnosis.

### Ethics

The study received a technical review from PIH and the Rwanda Research Committee. Ethical clearance was obtained from the University of Rwanda College of Medicine and Health Sciences, School of Public Health Ethics Committee and Harvard Medical School, and BWH granted an exemption for this study.

## RESULTS

There were 323 cases reviewed during the training segment, 374 cases during the technical workflow segment, and 256 cases during the testing segment (Table 1). The median age across the three segments was 44 years (interquartile range, 27 to 57 years). The majority of samples were from women (n = 687; 72%). Breast cancer was the most common diagnosis (n = 138; 34%), followed by cervical cancer (n = 90; 22%) Of the 953 total cases reviewed, 512 (54%) were malignant on slide review (Table 2). Of those, telepathology was able to confirm 194 (37%) malignant cases. Overall agreement was calculated for each phase by comparing malignant slide gold-standard case categorizations and corresponding confirmed telepathology categories of malignant, benign, infectious, or nondiagnostic. Agreement between slide and confirmed telepathology cases was 93% during the training segment and the technical workflow segment and increased to 97% during the testing segment (Table 2). There was no statistical difference in agreement between training and testing segments of implementation (Table 2). When penalty weights were applied to misclassified diagnoses (Table 3), similar results for agreement (96.1%) were found with the κ statistic for the final testing phase

**Table 1:**
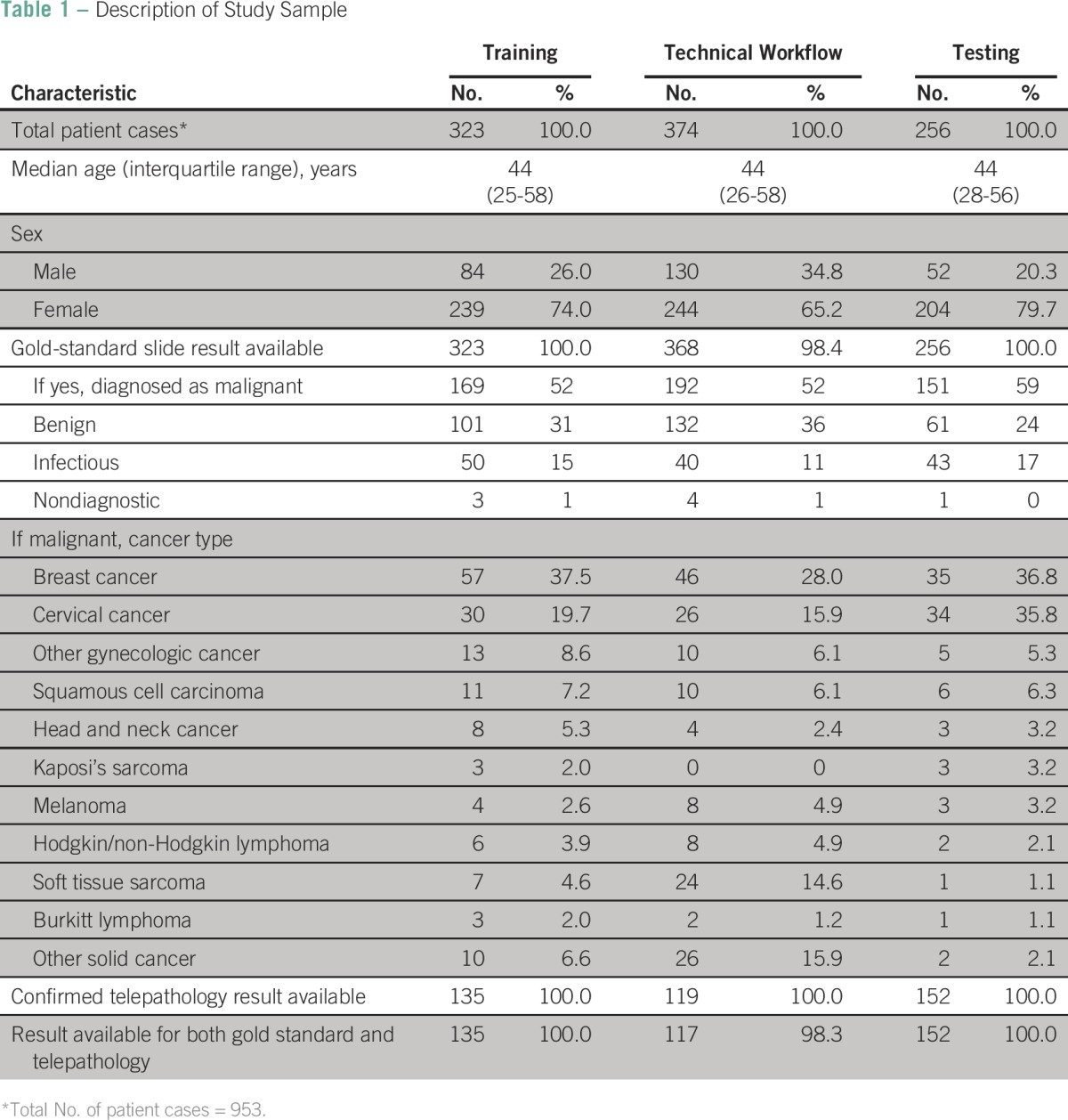
Description of Study Sample

**Table 2:**
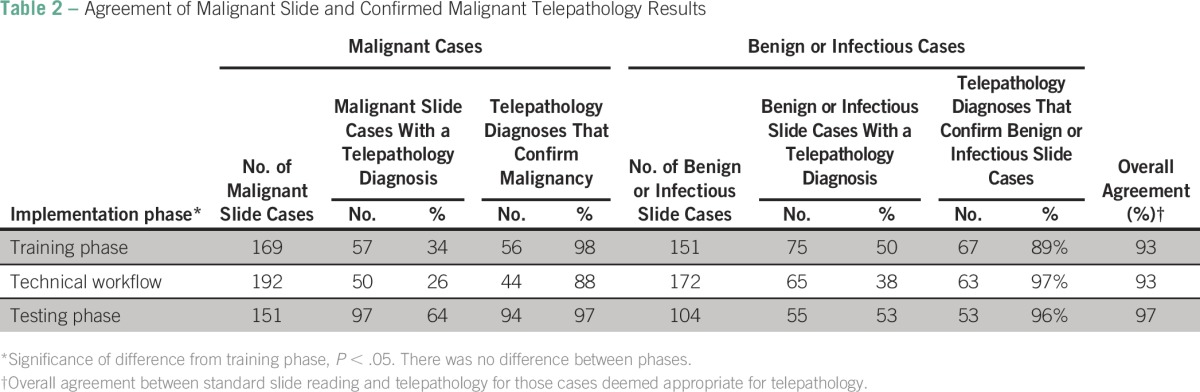
Agreement of Malignant Slide and Confirmed Malignant Telepathology Results

**Table 3:**
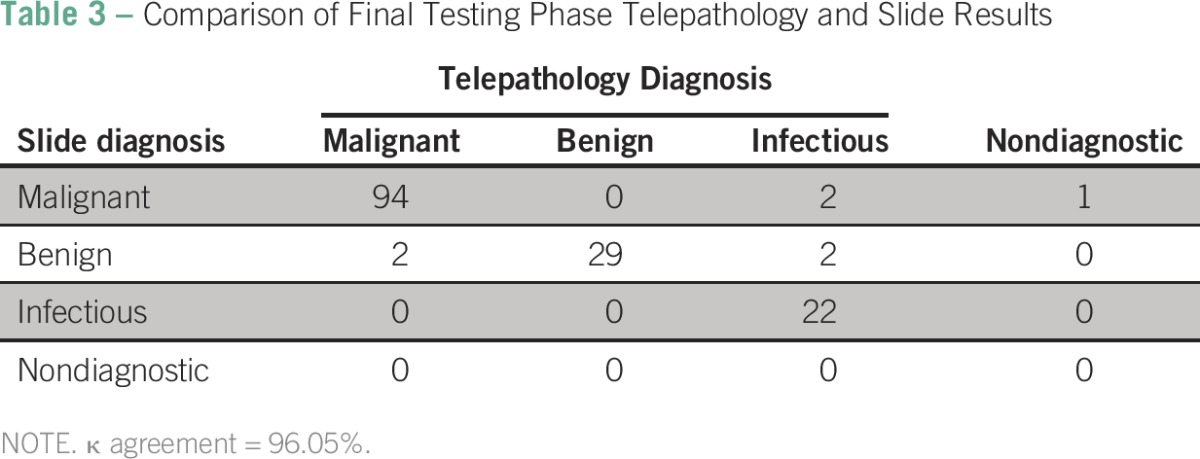
Comparison of Final Testing Phase Telepathology and Slide Results

The reasons for no final telepathology diagnosis are listed in Table 4. In the training segment, the comment “need entire slide” was a separate category and was the most common reason for triage (n = 91; 48%). “Immunohistochemistry needed” was also a common reason for triage across the phases (n = 84; 21%). By the final testing segment, the “need for an expert” was the most common reason for triage (n = 56; 54%). Image issues, such as “additional needed” and “poor quality,” remained a challenge at the close of the study (n = 22; 21%).

**Table 4:**
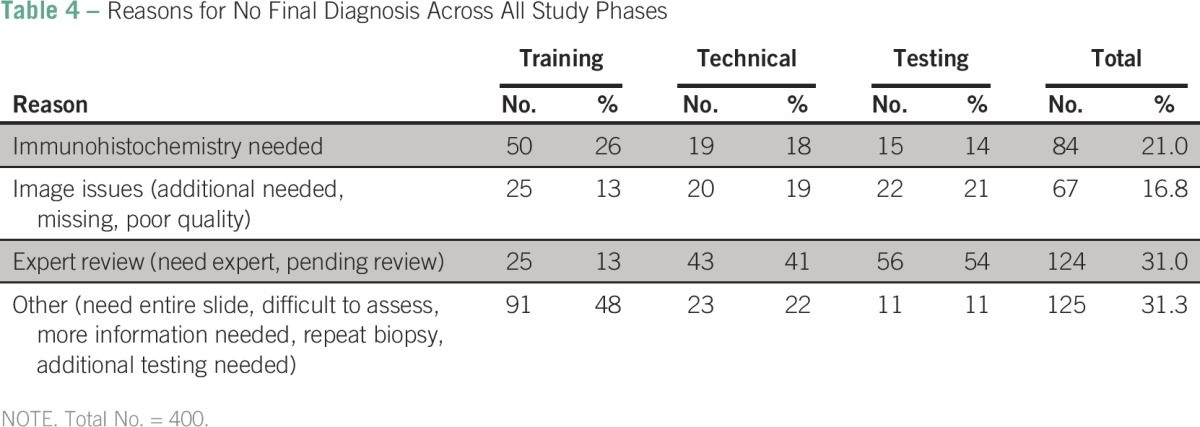
Reasons for No Final Diagnosis Across All Study Phases

## DISCUSSION

Offering accurate and timely pathology services in resource-limited settings is challenging because of the lack of trained pathologists and an inadequate laboratory and supportive care infrastructure. Our experience demonstrates that static digital telepathology can be established in resource-limited settings and can be a reliable and efficient means to provide both diagnosis and educational support to pathologists and histotechnologists in these settings. Important resources to make this approach successful that are based on our data include on-site immunohistochemistry for common lesions, inclusion of a wide panel of pathology experts for telepathology review, and continuing education and training for staff.

Our diagnosis accuracy between static image and glass-slide diagnosis of 93% to 97% was similar to other studies conducted in LMICs, which reported 94% to 98% concordance between telepathology results and conventional slide readings for cancer diagnoses.^9-11^ However, results from a multicenter study in the United States found that accuracy dropped when agreement was compared across multiple observers,^12^ and, in the future, we should study the interpathologist reliability for diagnosis that is based on telepathology images. One of the challenges of using a static image system is that diagnoses are subject to greater variability on the basis of image quality and adequacy of pathology sampling.^3,13^ For this study, bone marrow aspirates and hematologic malignancies were a priori triaged to Boston, because BWH pathologists felt that routine hematoxylin and eosin slides would be insufficient to diagnose these diseases. On-site flow cytometry and molecular testing in addition to immunohistochemistry are required to affect the turnaround time of these diseases. Image quality/quantity improved during the study, and rigorous adherence to protocols was the most effective way to ensure this. Some pathologic diagnoses simply require review of the entire tissue specimen, and this remained the most common reason for triage in our current phase of the study. The absence of immunohistochemistry at BCCOE during the study period was a major challenge to timely diagnosis and was the most frequent reason for telepathology inaccuracy. Similar challenges have been reported in Malawi and in a multicenter study.^4,12^

### Lessons learned for successful telepathology implementation

#### Determine the best telepathology system for your setting.

Dynamic imaging, a video consultation that keeps direct interaction between reviewers at the same time, and static imaging, which selects an adequate image from the original stained slide and sends these images to a reviewer via designated software, are the two most common types of telepathology. Although dynamic imaging has higher accuracy, the disadvantages are that it requires high internet bandwidth and high storage capacity and that individuals must be available on demand, often across time zones. A static imaging system uses a lower bandwidth and is less expensive, and we opted for this system with iPath because of low internet bandwidth and inconsistent connectivity.

#### Training and mentorship of local staff.

Pathology services in rural areas in Africa are almost exclusively staffed with laboratory technicians. Because these individuals may not have enough knowledge in histopathology, initial intensive training is required to equip them with basic skills in sample processing, staining, and image photography for telepathology. During grossing, sampling from the appropriate tumor locations is essential, and slide images must be obtained from diagnostic areas of the specimen. In our experience, this initial training can be completed in 3 to 4 months. However, skills training alone is not sufficient for making telepathology operational. In our study, when mandatory, rigorous protocols were not in place, the quantity and quality of cases uploaded decreased. Real-time feedback, constant communication, and team building were key interventions to improve the overall impact. Mentorship, ideally on site, is required, and we aim for semiannual mentorship visits from experts from partnering institutions.

#### Validation of telepathology results.

After full implementation of the telepathology services, results must be routinely validated to ensure quality. A less intensive check includes preparation and delivery of multiple slides through telepathology and assessment of the consistency of diagnosis between two images. A second validation process assesses the consistency of the diagnosis on the basis of the telepathology image compared with the diagnosis from an expert pathologist on the basis of the original slides. Ideally, this would be done locally, perhaps overlapping with the mentorship visits. However, this process of validation often could require delivery of the original slides to an international site for expert diagnosis. This international delivery option is resource intensive, but such validation is required to ensure an accurate telepathology-based diagnosis.

#### Scale-up to sustainability.

Diagnosis that is based on telepathology is not the long-term goal for BCCOE. Instead, it is an interim step while more sustainable solutions are developed. BCCOE has developed a long-term plan with BWH to include local staff training and, ultimately, a permanent histopathologist on-site. Training in pathology has occurred at the University of Rwanda since 2013, and this training will help ensure sustainability of the system at the national level. Telepathology will continue to be a valuable resource that gives local pathologists faced with challenging cases access to consultation from pathologists at academic cancer centers and that can serve as an ongoing training/mentoring tool.

In conclusion, our study demonstrates that telepathology service can be a reliable tool to support cancer diagnosis in resource-limited settings that have limited numbers of pathologists. Each pathology department has to test the type of system selected and validate its accuracy before it relies on telepathology as a standard approach for selected cases. The quality of the image produced as well as the selection of appropriate portions of specimen slides to photograph are important for maximizing diagnostic accuracy. Availability of immunohistochemistry staining performed locally also facilitates applicability of more cases for telepathology. Clear guidelines for triaging specimens suitable for telepathology need to be developed. In the future, we will assess the impact of the telepathology system on time from biopsy to diagnosis and will assess the start-up and recurrent costs of setting up such a system in rural African settings. After this validation, Butaro Hospital will develop a protocol for the routine use of telepathology. We recommend telepathology as a valuable resource for other programs in resource-poor settings that are attempting to improve access to cancer care.
